# Getting one’s bearings: a grounded theory study of fathers’ sense of security while attending the birth of their child

**DOI:** 10.1186/s12884-025-07969-3

**Published:** 2025-08-21

**Authors:** Therese Werner-Bierwisch, Sabine Metzing, Eva Kristina Persson, Claudia Hellmers

**Affiliations:** 1 Present Address: Department of Nursing Science, Faculty of Health, Witten/Herdecke University, Stockumer Strasse 12, 58453 Witten, Germany; 2 Research Group ‘FamiLe– Family Health in Life Course’, Witten and Osnabrück, Herdecke, Germany; 3 Niederrhein University of Applied Sciences, Krefeld, Germany; 4 Present Address: Faculty of Business Management and Social Sciences, Osnabrück University of Applied Sciences, P.O. Box: 1940, 49009 Osnabrück, Germany; 5 Department of Health Sciences, Lund University, P.O. Box 157, SE-221 00 Lund, Sweden

**Keywords:** Sense of security, Fathers, Pregnancy, Childbirth, Grounded theory

## Abstract

**Background:**

During the transition to parenthood, fathers face psychosocial changes that are accompanied by uncertainty and insecurity. Feelings of insecurity may affect fathers’ experiences of pregnancy and childbirth, adjustment to the paternal role, and bonding with their child. There is a need to better understand fathers’ experiences of security and the causal and constitutional factors involved. The aim of this study was to develop a theoretical model of how fathers’ sense of security is constructed during pregnancy and childbirth, with a focus on fathers who are present at birth.

**Methods:**

In line with Corbin and Strauss, a grounded theory methodology was used. The data were analysed using the principles of concurrent data collection and analysis, theoretical sampling, constant comparative analysis and memo writing. A total of 24 interviews were conducted 4 to 14 months after the birth of the participants’ last child during the 2016–2019 period.

**Results:**

Getting one’s bearings during pregnancy and childbirth emerged as the main category for fathers’ sense of security during pregnancy and childbirth. Six categories, ‘preparing for childbirth’, ‘agreeing with her decision’, ‘assessing and weighing risks’, ‘building a trusting relationship with professionals’, ‘taking appropriate supportive role(s)’ and ‘analysing the birth process’, underpin the core category and represent fathers’ strategies for achieving or maintaining orientation in changing situations. Fathers’ orientation strategies are influenced by factors such as personal skills, information, experiences, and the characteristics of professionals in either supportive or inhibiting ways. When fathers succeed in getting their bearings in particular situations, this success opens up possibilities for action and gives them a sense of control. In contrast, a lack of orientation may lead to fathers’ inability to act and may be accompanied by feelings of stress or worry about their female partner and child.

**Conclusions:**

Mothers’ wishes and needs and the competence of professionals are two essential sources of orientation strategies for fathers. Getting their bearings enables fathers to perceive themselves as competent and to cope with the uncertain and potentially challenging situation of childbirth. A better understanding of the factors that contribute to fathers’ sense of security can help professionals effectively support fathers’ need for security during pregnancy and childbirth.

## Background

In most Western European countries, it is common for expectant fathers to be actively involved in pregnancy and to support their female partner during childbirth [1–3]. The impact of fathers’ involvement on maternal and infant health has been identified in scientific research [4–10]. Despite the importance of their involvement for mothers and children, fathers face psychosocial changes in terms of their masculine identity and their relationship with their female partner [11–14]. Draper’s [11] transition theory describes fatherhood as a dynamic process that comprises physical, social, and emotional dimensions. The challenges associated with the transition to fatherhood have an impact on mental health and can cause uncertainty and insecurity in fathers, which can affect their experiences during pregnancy, childbirth, and the postnatal period [11, 12, 15, 16].

Social science studies characterise the concept of a sense of security as a cognitive perception of a physical state with the feeling of being protected [17–19] and as an individual emotion in which a person feels safe [20, 21], confident and free from anxiety [18, 22]. According to Porzsolt [23], feelings of security or insecurity result from an individual’s perception of his or her ability to cope with the challenges posed by the environment, including physical conditions, social support, structural factors, and social norms. Therefore, the factors that create a sense of security are relative and cannot be applied to all situations in life. Most social science research uses the terms ‘feeling secure’ and ‘feeling safe’ synonymously, suggesting that the concepts of security and safety are intertwined without a clear dividing line [17, 21].

In the context of maternity care, the concept of a sense of security has been studied mainly during the postnatal period, with a predominant focus on mothers’ experiences [24–30]. Few studies have addressed fathers’ sense of security in the postnatal period [28, 31, 32]. According to the findings of a previous integrative literature review, a sense of security depends on internal and external factors. For fathers, the internal factors include their emotional state, knowledge, and experiences, whereas the external factors include the health status of the mother and child and the characteristics and actions of health care professionals/midwives [16, 28]. When professionals involve fathers in the processes of pregnancy and childbirth and fathers receive consistent and relevant information and explanations, their sense of security is strengthened [28, 32–34]. In their qualitative study, Persson et al. [32] indicated that fathers’ postnatal sense of security emerges from their participation in and experiences of pregnancy, childbirth, and early parenthood.

Further studies of fathers’ childbirth experiences have shown that knowledge about childbirth increases fathers’ sense of security and is associated with a sense of control [35–38]. However, the opposite is also true: if fathers do not feel sufficiently involved and informed about what happens during childbirth, they feel a lower sense of security, which may be accompanied by a sense of lack of control [34, 37, 39].

Research on fathers’ sense of security remains limited and has focused primarily on the postnatal period. Few studies have examined the antenatal period and childbirth itself to comprehensively analyse the development of a paternal sense of security. The specific strategies that fathers employ to establish or maintain their sense of security and the factors that influence this process remain unclear. Moreover, existing research predominantly provides descriptive insights, whereas the relationships among the factors of paternal sense of security have rarely been explored.

To address this gap, this study explores how fathers’ sense of security is constructed and what strategies fathers use to achieve a sense of security during pregnancy and childbirth. The aim of this study is to develop a theoretical model of fathers’ sense of security in the context of childbirth, with a focus on fathers who are present at birth. Understanding how these fathers express and interpret their sense of security is essential for supporting their needs in the context of maternity care.

This study was part of a larger research project in which both parents were interviewed separately. The findings from the data on mothers are analysed separately.

## Methods

This study follows Corbin and Strauss’s grounded theory approach [40], which aims to develop theory grounded in data through exploratory analysis. Grounded theory is rooted in symbolic interactionism [41] and focuses on understanding lived experiences from participants’ perspectives [40, 42].

Among the methodological variations of grounded theory, this study applies the approach of Corbin and Strauss [40], which acknowledges the role of prior theoretical knowledge in sensitising researchers to data analysis. This approach also provides a structured coding paradigm to systematically develop categories and guide the analytical process.

### Sampling and participants

The participants were recruited mainly through gatekeepers and a published information leaflet. To ensure the heterogeneity of the group, the search focused on obtaining a variety of birth experiences in terms of the number of experienced births, the mode of birth and the birth setting. Based on theoretical sampling [40], in which the decision to select study participants is made simultaneously with the development of knowledge, the progressive analysis of the data and the development of further hypotheses led to a search for fathers with unique experiences. In the advanced stages of the analysis process, fathers with specific experiences throughout Germany were contacted via internet forums and the first author’s professional network. A total of 24 interviews with fathers were conducted between 2016 and 2019. Owing to data loss, two interviews could not be analysed, resulting in a final sample of 22 interviews that were included in the analysis. The fathers were between 28 and 50 years of age, and sixteen were first-time fathers. Almost all first-time fathers actively participated in antenatal education classes, accompanied their female partners to antenatal appointments, and were often present during the postnatal home visits by midwives. Table 1 shows the characteristics of the participants.

**Table 1:** Characteristics of the participants and interviews (*n* = 22)

Participant (ID number)	Age (years)	Nationality/ country of origin	Time of interview after childbirth (months)	Duration of interview (minutes)	Previous births	Birthplace	Mode of birth	Marital status	Occupation	Employment status	Highest educational attainment
1	41	German	6	13	Two	Birth centre	Normal vaginal delivery	Married	Nurse	Full-time employee	General Certificate of Secondary Education
2	34	German	12	23	Two	Hospital with freelancing midwife	Vacuum-assisted vaginal delivery	Married	Official	Full-time employee	Degree
3	31	German	9	13	Two	Home	Normal vaginal delivery	Married	Research assistant	Full-time employee	Degree
4	30	German	12	47	None	Hospital	Normal vaginal delivery	Living with partner	IT system developer	Full-time employee	Degree
5	28	German	8	23	None	Hospital	Normal vaginal delivery	Married	Professional driver	Full-time employee	General Certificate of Secondary Education
6	36	German	6	33	None	Hospital	Normal vaginal delivery	Married	Lawyer	Full-time employee	Degree
7	31	German	5	47	None	Hospital	Caesarean section	Married	Biologist	Full-time employee	Postgraduate degree
8	36	German	12	42	None	Hospital	Vaginal breech delivery	Married	Insurance company employee	Full-time employee	Postgraduate degree
9	50	German	8	40	One	Hospital	Normal vaginal delivery	Married	Graphic designer	Full-time employee	A-Level
10	39	German	12	42	One	Home	Normal vaginal delivery	Married	Master gardener	Full-time employee	Degree
11	35	German	7	44	One	Home	Normal vaginal delivery	Married	Molecular biologist	Full-time employee	Postgraduate degree
12	35	German	12	45	One	Home	Normal vaginal delivery	Married	Industrial engineer	Full-time employee	Degree
13	30	German	8	93	None	Hospital	Vacuum-assisted vaginal delivery	Living with partner	Event technician	Full-time employee	A-Level
14	38	German	9	47	One	Home	Normal vaginal delivery	Married	IT specialist	Full-time employee	A-Level
15	34	German	14	37	One	Hospital	Elective caesarean section	Married	Economist	Full-time employee	Degree
16	41	German/Romanian	8	30	None	Hospital	Vacuum-assisted vaginal delivery	Living with partner	Insurance broker	Full-time employee	Degree
17	34	German	6	52	None	Hospital with freelancing midwife	Caesarean section	Living with partner	Engineer	Full-time employee	Degree
18	37	German	6	37	None	Hospital	Normal vaginal delivery	Married	Financial broker	Full-time employee	A-Level
19	32	German	4	71	One	Home (unassisted birth)	Normal vaginal delivery	Married	Media consultant	Full-time employee	Degree
20	37	German	11	43	None	Hospital	Caesarean section	Married	Engineer	Full-time employee	Degree
21	33	German/Romanian	4	102	None	Home (unassisted birth)	Normal vaginal delivery	Living with partner	Molecular biologist	Part-time employee	Degree
22	28	German	6	43	None	Hospital	Emergency caesarean section	Married	Engineer	Full-time employee	Degree

### Data collection

Data were collected from participants in three German federal states, with interviews conducted between 4 and 14 months after the birth of the (last) child. The later start of data collection was considered reasonable, as coping with and adjusting to the role of father usually occurred during this time [43]. Semistructured interviews were conducted to structure the open narrative space. In addition, the interviews were characterised by a series of predefined topics that were intended to give the interviewees direction when narrating their experiences [44]. The interviews were conducted mainly in the participants’ homes or in public places of their choice and lasted between 13 and 102 min. The interviews started after the fathers provided written consent to participate in the study, which was sent to them in advance with the study information. The interviews started with the following narrative stimulus: ‘What were your expectations for the upcoming birth?’ or ‘What was important to you about the upcoming birth?’. To ensure the greatest possible openness, the order of the questions was not predetermined but based on the subjective relevance assigned by the interviewees. It was important to support the interviewees’ flow of words with appropriate question forms. Towards the end of the interviews, the interviewees were again allowed to determine relevance and to comment on the course of the interview [44]. For this purpose, the following open-ended exit questions were asked: ‘In conclusion, is there anything else you would like to add about your sense of security?’ or ‘If you look at your story again now, what aspects made up your sense of security?’ As the analysis progressed, some of the questions in the guide were clarified or supplemented to reflect the evolving theoretical relevance of the concepts [40]. The first author developed the interview guide and conducted all the interviews. The interviews were recorded and transcribed verbatim, and the first author reviewed each transcribed text to ensure accuracy. Two interviews could not be included in the data analysis due to the loss of voice recording data.

### Data analysis

The 22 interview transcripts were analysed using concurrent data collection, constant comparative analysis, and memo writing until data saturation was reached [40]. In line with Corbin and Strauss’s [40] grounded theory approach, the analysis followed three coding steps: open coding (identifying phenomena), axial coding (relating codes to each other), and selective coding (developing a core category) [40].

Initially, the transcripts were read to gain an overview and to identify relevant textual units. These were coded line by line while remaining close to the participants’ original expressions. During axial coding, the relationships between codes were examined using the coding paradigm, which included conditions, context, action strategies, and consequences [40]. Hypotheses about category relationships were tested and refined through further data analysis. Selective coding focused on elaborating the core category, ‘getting one’s bearings’, and validating it through systematic integration with other categories and theoretical saturation. Once saturation was achieved, a final validation step was conducted through a telephone interview with the last participant to further confirm explanatory relevance.

The analysis process was documented in theoretical memos, and theoretical sampling was used to refine the emerging hypotheses. To ensure rigour, the first author conducted the primary coding, followed by discussions within the research team. The final categories were collaboratively reviewed and refined. Data analysis was supported by MAXQDA 12 and 18 and MAXQDA Plus 2020.

## Results

### Overview of the theoretical model

**Fig. 1:**
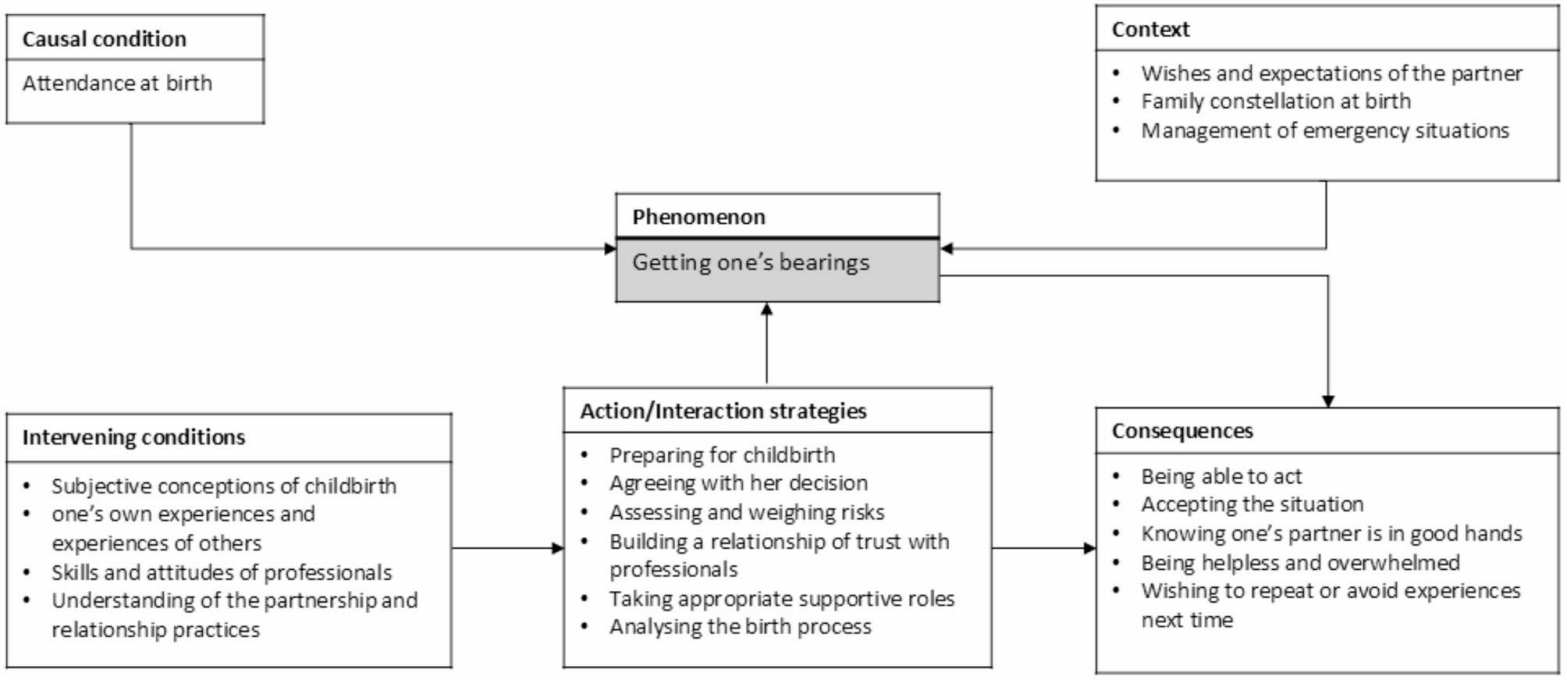
The theoretical model of fathers’ sense of security in the context of childbirth

As expected in a grounded theory approach, the theoretical model represents an emerging theoretical idea and a conceptual framework developed from the data. The model illustrates how fathers’ sense of security in the context of childbirth is shaped by various interacting factors and processes. In line with Corbin and Strauss’s (2014) framework, this model captures the relationships between the conditions, strategies, and consequences that influence fathers’ experiences of security during childbirth. Figure 1 presents a visual representation of this theoretical model.

With an upcoming birth as a causal condition, the support of female partners places different demands on fathers, which they attempt to manage. Getting one’s bearings in these situations was identified as the core category in the data on fathers’ sense of security during pregnancy and birth. Fathers use different action/interaction strategies at different times to achieve or maintain this state in changing situations. These include ‘preparing for childbirth’, ‘agreeing with her decision’, ‘assessing and weighing risks’, ‘building a relationship of trust with professionals’, ‘taking appropriate supportive role(s)’ and ‘analysing the birth process’. Fathers’ application of these strategies is determined by a variety of intervening conditions that facilitate or inhibit their strategic actions. The most important resources for shaping fathers’ orientation strategies are their subjective conceptions of childbirth, birth experiences or the experiences of others, the skills and attitudes of professionals, information, and fathers’ partnership-based relationship practices. In addition, fathers’ ability to orient themselves is embedded in a context. Corbin and Strauss [40] define context as a set of characteristics that belong to a phenomenon and within which action strategies take place. Within the framework of this theoretical model, the wishes and expectations of the female partner, the management of emergency situations and the family constellation during childbirth care form the case-specific context. If fathers succeed in getting their bearings, this success opens up the possibility for them to act and accept the situation as it is. Their belief that their female partner is in good hands with the caregivers helps them find a way to deal with the situation, especially when they see no options for action for themselves. If fathers do not get their bearings in a situation, they may feel helpless and overwhelmed. The consequences of birth experiences ultimately shape the approach to future birth situations. Fathers want to repeat the experience after a positive birth experience or avoid negative experiences as much as possible.

A detailed description of the theoretical model, including quotations from the fathers’ statements, follows below.

### Getting one’s bearings

The ability to get one’s bearings in a given situation, understood in this study as the active process of orientation, is central to fathers’ sense of security and is closely linked to their understanding of the situation and what needs to be done. The situation is viewed as the epitome of what fathers are currently experiencing. One father explained, ‘I think that by simply being more involved, by seeing what is happening now, that you can do something for them yourself, you feel secure, you feel more secure’ (P2).

In this context, getting one’s bearings does not refer exclusively to the birth situation; it is presented in the data as a continuous process that begins during pregnancy and continues beyond birth. The data indicate that fathers actively engage in orientation strategies to facilitate and maintain this ongoing process. During pregnancy, fathers seek orientation strategies by preparing for the birth and actively participating in the decision regarding the place of birth. One father stated, ‘I already want to prepare myself for what will or can happen so that I can cope with it and not get lost in the situation. That is important for my sense of security’ (P17).

In the dynamics of childbirth, getting one’s bearings is not a static, permanent state; rather, it is a fragile process that can be disrupted by unexpected developments. If fathers are not familiar with new events as they occur, they may lose their bearings. To maintain their bearings, fathers constantly compare the specific circumstances of the birth

I had the feeling that I was constantly in observation mode, not just to check what was happening but because the situation can change quickly. So, I wasn’t under tension but somehow had everything and everyone on my radar to be able to understand what was happening, to be able to be fully involved. (P9)

### Context

Fathers’ ability to get their bearings is embedded in a context. Below, various contextual aspects that can be derived from the data on fathers are presented. As case-specific characteristics, contextual factors are the concrete, situation-specific attributes of getting one’s bearings.

### Wishes and expectations of the partner

The partner’s wishes and expectations regarding childbirth and care influence how fathers develop their orientation strategies. Fathers align their actions to provide support, whether through presence, through emotional reassurance, or by taking on specific roles. While some feel a strong sense of responsibility to meet these expectations, others express uncertainty about their role. Some fathers express a strong commitment to aligning their actions with their partner’s wishes and see it as their responsibility to meet her expectations. As one participant described, ‘I wanted to support her wishes and expectations as much as possible. What mattered to her became my goal, and I worked towards it and adjusted my actions accordingly’ (P2).

### Family constellation at birth

The organisation of childcare and family involvement around childbirth influences how fathers get their bearings in their supportive role. When childcare arrangements are secured, fathers can focus on their partner’s needs. However, if responsibilities remain unclear, they may have to divide their attention between different roles. While some fathers welcome the presence of extended family members, others find their presence disruptive, particularly when it makes their own role uncertain. In some cases, they struggle to position themselves in the support process when other family members take an active role, which sometimes alters the couple’s dynamic. One father described feeling pushed aside when his partner’s mother became too involved: ‘And I actually felt […] that I wanted to protect (woman’s name) a bit from this “doer energy”. Her mother, to my taste, was a bit too quick and too active in that moment. […] But actually, it threw me out of my role’ (P21).

### Management of emergency situations

Complications during childbirth and the dynamic responses of caregivers serve as additional contextual conditions for fathers’ ability to get their bearings. Effective communication and the perception of professional competence help fathers maintain a sense of security and situational awareness and reduce anxiety and uncertainty. In contrast, when fathers are excluded from emergency management, their ability to get their bearings is significantly impacted. This is particularly evident during emergency caesarean sections. In Germany, fathers are usually not allowed in the operating room and must remain in the delivery room. The sudden isolation from their partner and professionals can trigger an emotional crisis, as both key orientation figures are abruptly removed. One participant described his distress vividly: ‘[…] I was pacing around the room, feeling really stressed out because everyone had left and I could no longer see what was going on’ (P20).

### Action/Interaction strategies

According to the interviews, six essential orientation strategies that fathers use during pregnancy and in the birth situation to find their way in certain situations and to open up possibilities for action can be identified. The action/interaction strategies identified from the data are presented in more detail below.

### Preparing for childbirth

Being prepared for childbirth played an essential role in helping all the interview participants cope with the demands of childbirth. The fathers’ motivation to prepare for childbirth was influenced by factors such as their perceptions of childbirth, their resources, and their partner’s needs or wishes for support.

Three levels of preparation were identified in the data: at the cognitive level through knowledge acquisition, at the practical level through targeted preparation activities, and at the mental level by coping with the upcoming situation. The levels of preparation are interrelated and should not be understood as separate processes. The preparation strategies are presented in three subcategories: informing yourself, preparing mentally for the birth and preparing for an emergency.

#### Informing yourself

To prepare themselves for the birth, fathers use various sources of information, such as talking to their partner, attending antenatal education classes or receiving relevant information when accompanying the mothers to antenatal visits. In particular, exchanges with the female partner play an important role. These exchanges are essential to prepare them for their supportive role, regardless of whether the fathers have had any previous childbirth experience. One father emphasised, ‘For me, the crucial thing was that she told me what she needed from me at that moment’ (P10).

The data also show that the level of trust in one’s partner or caregivers influences the intensity of information seeking. If fathers (even in the case of a first birth) have basic trust in the natural birth process or in their partner’s ability to give birth, they feel less compelled to obtain specific information about the birth and the processes involved. One father explained, ‘But the body does it, and that’s why I had […] no interest in reading or understanding things or parameters or values or anything’ (P21).

Trust in the obstetric expertise of professionals also affects the level of information and leads fathers to believe that their partners will be adequately cared for without seeking information about birth procedures. The certainty of adequate care for the mother and child enables fathers to relinquish responsibility and control to the caregivers. In addition, the content regulates the need for information. In particular, a detailed description of birth complications or labour pains can be threatening and frightening for some fathers. One father stated, ‘However, that’s just this double-edged sword. Too much information also plants pictures in your head that you don’t want to have’ (P11).

To protect themselves and to avoid being emotionally overwhelmed, some of the interviewees made conscious decisions to avoid obtaining knowledge about childbirth.

#### Preparing mentally for the birth

While almost all the interviewees mentioned preparing for the supportive role and wanting an overview of possible birth processes, only a few consciously addressed mental preparation. Implicitly, however, there was a specific need for an inner confrontation with the topic of birth owing to the new situation and the associated demands placed on them in their role. In some interviews, childbirth was seen as a situation that ‘has to pass’ or as a situation that could be managed ‘because you go in there with the right attitude’. The quotes reveal a challenging characteristic of the childbirth situation that must be overcome with a positive mental attitude. Beyond acquiring factual knowledge, some fathers actively worked on developing an inner mindset to manage stress and uncertainty during childbirth. This included strategies to stay calm, maintain emotional stability, and adapt to unexpected situations. One interviewee described how attending a childbirth preparation course helped him approach the birth with greater confidence and emotional readiness:

But now I think it wasn’t wrong at all to take a childbirth preparation course to get ready for the phase before birth. Even though things turned out differently during the birth, it […] helped me go into it with a better feeling. (P16)

The data show that some form of mental preparation is particularly important for fathers who are faced with a new situation and who have no direct experience to draw on.

#### Preparing for an emergency

Safe medical care for mothers and children was a high priority for almost all the fathers. Against this backdrop, it was essential for the fathers that the birth took place in a safe space to ensure timely emergency care for the mother and child. Nevertheless, the fathers tried to plan for what they would do in a worst-case scenario, and they took practical steps to be prepared. This was done regardless of the chosen place of birth, although the place of birth influenced the level of preparation. Emergency scenarios were sometimes vaguely defined in the data with references to situations described as ‘something bad’, something ‘going wrong’ or ‘panic’. In this context, an emergency for the fathers did not necessarily have to be accompanied by visible danger. Even a situation that was not assessable and clear, in which a possible threat to the mother or child could not be ruled out, could be classified by the fathers as an emergency and usually required action. The fathers saw their contribution as called for when the professionals were not (yet) responsible or as a complementary measure to ensure preparedness in case of an emergency. This was particularly relevant in cases of planned home births attended by midwives, where fathers took proactive steps to ensure that they could react quickly if medical intervention became necessary. One participant described how he prepared for such a scenario:

And if it doesn’t work, we just have to think through different procedures in advance. That means the phone has to be there, the emergency numbers have to be available, the nearest delivery ward has to be informed. […] That means I also drove the routes beforehand and said, ‘Okay, how long do I need from here to there?’ […] Yes, that just gave me a better feeling. (P12)

By preparing for potential emergencies, the fathers ensured that they had concrete options for action if necessary. Their primary concern was to be able to respond effectively and in line with their expectations of support and responsibility.

### Agreeing with her decision

As the fathers attributed the central role in the birth to their female partner, they attributed decision-making competence to her as well. Therefore, the fathers viewed their tasks in the decision-making team as supporting their female partner in her choice of birthplace or in the decision-making processes. They acted as discussion partners, companions or co-decision-makers who, together with their partner, reflected on the advantages and disadvantages of different birthplaces. However, to support the female partner’s preference or decision, the guarantee of safe care for the mother and child was an essential prerequisite for the fathers to ultimately agree to their partner’s preferred place of birth. If the mother’s preference did not match the father’s idea of a safe birth environment, this mismatch could lead to a lack of understanding on the part of the father and, thus, to conflict between the partners. One father described how differences in perceptions of safety influenced discussions with his partner about the place of birth:

For me, the safest thing in my head was actually in the hospital or the delivery room with midwives, where you can always call in a doctor […]. I just had the feeling that (…) it always has to be like this, that someone is there who knows about medicine. And that’s where we disagreed at first, which led to many discussions. (P11)

### Assessing and weighing risks

Assessing and weighing of risks are related to two different points of reference: on the one hand, the paternal perception of risk for the mother and child; on the other hand, an assessment of the father’s own vulnerability in terms of emotional or physical strain in relation to the upcoming birth. When assessing the risk to the mother and child, the participants did not rely exclusively on the risks identified in the mother’s medical history or in the care context. Rather, they relied on the possibility of a risk situation that could arise during the birth. The data suggest that the perception of possible risks for the mother or the child was closely linked to the fathers’ conceptions of childbirth. When birth was perceived as an event that was fundamentally associated with risks to the mother and child, most fathers preferred a clinical birth setting, which was the only option where potential birth risks could be addressed under medical supervision. One participant described this perception as follows:

So, on the one hand, it is a risk, and on the other hand, of course, it is first and foremost a very beautiful event. But it could lead to some kind of complications, and then, of course, you are well taken care of if you have the best medical possibilities available. (P20)

Previous childbirth experiences also influenced the fathers’ risk perceptions. For example, fathers who had positive birth experiences considered the likelihood of complications and, thus, the risks of childbirth to be lower. The experience of a normal birth without complications was used as the starting point for the new risk assessment. This was expressed as confidence to the point of being convinced that the birth would be unproblematic again. However, the fragility of this belief became apparent when the mother did not prefer a clinical setting and, instead, preferred an out-of-hospital birth environment. In this case, the fathers needed external validation and included the expertise of obstetricians in their risk assessment. The clarification or exclusion of risks by obstetric professionals was a crucial basis for paternal approval of an out-of-hospital birth. One participant described how reassurance from an obstetrician influenced his confidence in this decision: ‘The obstetrician also did the one or two ultrasound tests that are mandatory. Everything was fine. There was no reason at all to have any other or different thoughts there’ (P3).

The fathers were reluctant to address their own vulnerability, as they wanted their partner to focus on the birth without worrying about them. Instead, they kept emotional or physical strain to themselves or downplayed it. One father explained, ‘I mean, I didn’t want to burden her with my worries that I might have circulation problems. After all, I have to be there for her, and she shouldn’t have to take care of me as well’ (P16).

### Building a relationship of trust with professionals

During childbirth, the fathers expected obstetric competence and integrity from the staff who cared for their partner and child. Therefore, the fathers entered into a relationship of trust with professionals, knowing that their partners would receive appropriate care and that they could rely on the professionals’ expertise in unpredictable situations. However, the fathers were unable to assess the competence of the caregivers because they lacked obstetric expertise. Therefore, the lack of verification made the expectation of appropriate care a matter of faith, leading to a sense of trust and confidence in the birth situation.

And in principle, I had this confidence with the feeling that the hospital we chose is just what we want to have, […] I always had the confidence that the staff make these decisions in a way that suits everybody. (P22)

The fathers’ perception of the caregivers’ obstetric competence played a significant role in building trust. The establishment of trust was based on the directly perceived trustworthiness of the caregivers, and competent and self-confident impressions were essential. In addition, commonalities in the attitudes of the caregivers were sought. One father stated, ‘Yes, with an appropriate looseness but also in a hands-on way. […] So, not always calming things down but also addressing them, and that was what appealed to me and my wife, the way she worked’ (P3).

The competence of the actors in charge, which was necessary for the desired care, was not only attributed to them on the basis of trustworthy performance but also assumed due to their profession. One father remarked, ‘At the end of the day, it’s really their job, what they do every day, and you assume it will be correct’ (P15).

Owing to the trust placed in them, the professionals enjoyed a certain immunity; thus, the fathers did not always notice treatment measures that were unfavourable for their female partner or behaviour that was critical for trust. As a result, the fathers rarely considered the failure of medical standards in the care situation. One father explained, ‘Of course, I wasn’t aware or, at least, not in that situation that the epidural probably didn’t work as it should have. You don’t even think about something like that, that it won’t work now’ (P6).

Our data analysis did not reveal specific differences in the development of trust among fathers with home birth experience compared with experience in clinical settings. Rather, trust was shaped primarily by the quality of the fathers’ experiences with the professionals involved, regardless of the birth setting.

### Taking appropriate supportive roles

Fathers support their partners during childbirth in many ways. As active helpers, they hold and support their partner during labour, reassure her or motivate her to continue. When active support is not required or not desired by the partner, fathers act as ‘stand-by’ supporters, listening to her signals and returning to the active role when instructed to do so by the midwife.

Supporting their partner during birth was taken for granted by almost all the interviewees. Being able to take on a supportive role enabled the fathers to find a place in the situation and contributed to their sense of getting one’s bearings. One father stated, ‘My task was to be there for her, to be with her and to give her this space. And for me, everything was clear, and it was like a direction for me’ (P21).

The fathers understood their support not only as the fulfilment of the tasks assigned to them but also as a basic attitude towards their partners, which was an aspect of their everyday relationship. In addition to the expected benefits for the mother, the supportive role provided an opportunity to act, which gave the fathers a sense of control in an unpredictable and uncertain birth situation.

So, it’s kind of like, you’re in it, you feel like you can always influence something, you/I don’t know if it’s a glass of water you’re handing or whatever, you’re kind of more in it, and you also kind of feel like you can be more involved, that you have a task and a place in what’s going on, that you’re more in control of it. (P2)

Taking on a supportive role meant, on the one hand, accepting role attributions from outside, such as from the partner or from professionals. Role attributions by professionals primarily took place in the context of antenatal classes and appeared to be particularly relevant for fathers who lacked prior birth experience. These courses provided the fathers with an understanding of potential support strategies and helped them shape their own role during childbirth.

On the other hand, the fathers brought with them role expectations derived from their conceptions of childbirth. In addition, they used different self-concepts or previous biographical experiences to outline their role profile in the upcoming birth situation. In the context of childbirth, the fathers assumed a fundamental ‘natural’ role asymmetry against the backdrop of their subjective conceptions of childbirth, which resulted from the attribution of the ‘main role’ to the woman giving birth. The fathers’ role was usually limited to supporting activities, which they saw as a ‘secondary role’. One father remarked, ‘So, because it was clear to me that the focus is on the wife and child, I could only help my wife by supporting her, fetching something and holding it and talking her down’ (P22).

Mothers communicated their role expectations by clarifying their needs and giving the fathers a better idea of what support was most helpful during the birth. If there were supportive factors that needed to be improved for the upcoming birth, the fathers’ supportive role was optimised or supplemented with the desired aspects. If there were factors that needed to be improved for another upcoming birth, the supportive role was supplemented with the desired aspects or improved if necessary. One father explained, ‘Yes, I was a bit more than just present for the second time. It was also quite clear that I was the communication channel to the medical staff […]’ (P9).

However, the implementation of this role was not always without difficulties. In particular, restraint at the woman’s request seemed particularly difficult for the fathers, who relied on concrete tasks as a primary coping strategy in challenging situations. In addition, there was the particular challenge of appearing calm and relaxed on the outside while hiding the inner tension and nervousness that resulted from their passive role. Therefore, the fathers tried to balance their inner emotional state by distracting themselves with complementary tasks.

She withdrew a lot and said, ‘I want to breathe through this now,’ and well, then I became the nerd. When she asked me, ‘How long are the contractions?’ I immediately handed her two A4 pages and said, ‘Here, this is the last hour– what exactly do you want to know?’ […] So that was my task. (P12)

### Analysing the birth process

The mothers and professionals were important sources of guidance for the fathers and represented a navigation system for them. When the fathers interpreted and reacted to the situation, they drew on a variety of references, such as everyday concepts or the birth experiences of others. The patterns of action used in the strategy helped the fathers obtain an overview of the situation so that they could assess the possible course of the birth or understand other changes in the birth process as well as the actions that needed to be taken. In addition, assessing the situation enabled the fathers to identify new options for action when they seemed appropriate and helpful.

#### Interpreting the reactions and behaviour of the female partner

Orientation towards the female partner was based on her behaviour and statements about physical sensations, which the fathers constantly interpreted. While first-time fathers used knowledge from antenatal classes and information about birth from their social environment, experienced fathers could draw on their own previous birth experiences and rely on the woman’s physical sensations.

Assessing the possible start of labour was particularly important for the fathers, as it involved specific tasks for them, such as travelling to the clinic or calling the midwife in time for the birth. As the fathers usually did not have access to the expertise and assessment of caregivers at the time of possible labour, they felt more responsible for their partners. The first stage of birth was managed differently depending on the resources available. If the parents already had experience of childbirth, experienced fathers trusted their partner’s physical perception and took their cue from her physical feelings and assessment of the situation.

So, it was clear to me that I had to orient myself to her to make sure that it was really going to start now. So, even if it’s the second time, a man somehow can’t estimate when the child will really come. (P14).

#### Interpreting professionals’ communication and behaviour

Orientation towards professionals took place in two ways: through the information received and by observing and interpreting the reactions and actions of the professionals. Orientation towards information was strongly dependent on the communication culture of the professionals.

Communication geared towards the needs of fathers helped them orient themselves in the birth situation and accept circumstances beyond their control. In rapidly changing birth situations, the fathers needed explanations and instructions to find their place and not get in the way of the caregivers.

That was quite good, and he said, ‘Please don’t look over the curtain, and when we go into the operating room, don’t look at your wife. Sit at your wife’s head and talk to her in an encouraging way; it’ll be fine. We’ll show you exactly where.’ (P17).

Friendly and positive communication also helped to create a positive atmosphere. If the information provided was inadequate, the birth situation remained unclear to the fathers. Although the fathers perceived the communication to be inadequate, they did not necessarily actively request more information. On the one hand, the fathers did not want to disturb the caregivers. On the other hand, they attempted to assess the situation on the basis of the professionals’ nonverbal signals and actions.

The elements of nonverbal communication included the facial expressions, gestures and physical reactions of the midwives and obstetricians. Observing and interpreting these reactions and actions enabled the fathers to check the content of verbal communication for congruence with the professionals’ reactions and to gain an impression of whether the birth situation was proceeding normally or whether possible complications were emerging. If the professionals’ behaviour and actions were perceived as calm and routine, they indicated a normal, unremarkable situation. On the other hand, nervous and hectic gestures or worried facial expressions were interpreted as indications of possible complications during the birth. One father recalled, ‘The doctor looked at it [cardiotocography] and was a little bit worried […]. Definitely something that unsettled me, where I thought, hopefully nothing would happen’ (P4).

## Conditions

From the data, numerous influential factors and conditions were identified that may influence paternal orientation strategies. First, attendance at birth is a causal condition for the central phenomenon. The intervening conditions that may have a beneficial or inhibitory effect on paternal strategies include *subjective conceptions of childbirth*,* one’s own experiences and the experiences of others*,* the skills and attitudes of professionals* and the *understanding of the partnership and relationship practices.*

### Causal conditions

#### Attendance at birth

Attendance can be attributed to different motivations, but it is intrinsically based on a personal attribution of meaning or extrinsically based on the perceived importance of the female partner or a social expectation. Intrinsic motivation is reflected in the data on the desire to experience childbirth together as partners and in identification with the role of father. Extrinsic motivation can be seen in the nature of the demands made on the fathers, either because of the obligation to the female partner, to which there is no alternative, or because of the social expectations that the fathers perceive and that can be accompanied by social disapproval if not fulfilled. One father noted, ‘My wife always insisted on it […]. And I also realise that it’s hard to avoid it’ (P6).

### Intervening conditions

#### Subjective conceptions of childbirth

The fathers’ conceptions of childbirth were multifaceted and sometimes ambivalent. Their perspectives reflected both the possible consequences for the mother and child and their own ways of coping with the situation. Three interconnected aspects shaped their understanding: childbirth as an existential experience, as an uncertain and unpredictable event, and as a phenomenon positioned between naturalness and risk.

Several fathers described childbirth as a deeply existential experience and emphasised its profound impact on human existence. Their descriptions ranged from seeing birth as a transformative event that brought new life to perceiving it as a physical borderline experience involving existential threat. This sense of risk materialised in concerns about physical integrity, potential complications, or even death. To mitigate these risks, the fathers emphasised the need for optimal and safe care. One father stated, ‘Most of all, we were concerned that it was kind of safe, that there was a high probability that everyone would survive’ (P13).

At the same time, the fathers highlighted the uncertainty and unpredictability of childbirth. Many described how the wide range of possible scenarios made it difficult to anticipate the process and outcome. The unknown nature of childbirth contributed to feelings of insecurity and required the fathers to develop strategies to manage this challenge. One father explained, ‘My not knowing what is going to happen, and in that not knowing, there is an uncertainty’ (P21).

Another central aspect of the fathers’ conceptions was the tension between naturalness and risk. Childbirth was described as a biological and natural process but one that remained inherently linked to the possibility of complications. The fathers acknowledged that while birth is a natural event, it does not necessarily proceed without difficulties. One father remarked, ‘So, for me it is first and foremost a biological process, a natural process, but it doesn’t always have to function so smoothly naturally’ (P18).

Despite this awareness of potential risks, some fathers, particularly those with experiences of out-of-hospital births, viewed childbirth as a self-regulating and naturally designed system that relied on biological mechanisms that have ensured human reproduction for millennia.

Even before modern medicine, […] billions of women in the world were able to do that. […] Otherwise, nature has set it up pretty well with hormones in different forms, which trigger the processes of pregnancy and then the induction of birth and so on. (P19)

### One’s own experiences and the experiences of others

The fathers’ own previous experiences, as well as those of others, were valuable resources that were incorporated into their strategies. Experiences from previous births contributed to a sense of familiarity and ‘routine’ in the new birth. One father explained, ‘The second time, [partner’s name] knew better what was going to happen. I knew better what was going to happen’ (P9).

Biographical experience was another resource for the fathers that was used to emotionally prepare for the birth. For example, the fathers used strategies that they had previously tried in the professional context, which enabled them to successfully deal with stressful situations and to control the inner tensions that arose during the birth. One father stated, ‘So, this radiating outwards, this calmness, which of course you also have a lot from your profession […] that was the same at birth, not consciously, but simply, why should you panic?’ (P12).

The experiences of others served as a substitute for some fathers’ lack of personal experience of childbirth. In this context, the experiences of others were used with different intentions, both for first-time fathers and for experienced fathers. For example, the experiences of family and friends were used to decide on a suitable place for the birth or to choose a qualified midwife for postnatal care.

### Skills and attitudes of professionals

The assessment of the competencies of midwives and obstetricians was based on specific behavioural characteristics and attitudes, such as experience, self-confidence, a calm demeanour, routine, transparent and open communication, restraint, and goal-oriented behaviour. In particular, the fathers equated self-confidence, especially when combined with the older age of the midwives in charge, with obstetric experience and competence.

The midwife who was with us at the end, I would say she made a very EXPERIENCED impression. I mean, she was an older lady. That’s probably why you’d say she had a lot of experience, but she was also SO UNBELIEVABLY calm. (P8)

### Understanding of the partnership and relationship practices

For the fathers, their understanding of the partnership and the relationship practices associated with it provided additional resources for their orientation strategies. In almost all the interviews, there were moments in which the fathers switched from their personal experience to a shared ‘we’ perspective, thus transforming the experiences into a couple’s experience. In the narrative passages, the ‘we’ perspective was linked to events experienced as a couple, and it reflected the partner’s horizon of experience. One father explained, ‘We also experienced the pregnancy together. And then it was clear that we also wanted the birth together, and we would do everything to make that possible’ (P22).

The interviewees drew on certain features of their partnership relationship model to shape their strategies. Two main features were identified in the data that characterised the relationship model of the study participants: *being in agreement with each other* and *being familiar with her perspectives*.

#### Being in agreement with each other

In addition to viewing the pregnancy and childbirth as a shared experience, the fathers relied on coping strategies that they had previously applied in their everyday partnership to navigate new processes and optimise forthcoming courses of action. In this context, they placed the issue of agreeing on decisions related to the birth within the realm of partnership responsibility, as they considered agreement to be a fundamental element of a relationship. One father remarked, ‘We basically agree on the elementary things. […] We had a big common denominator and didn’t have to make massive compromises to somehow approach each other’ (P19).

#### Being familiar with her perspectives

To cope with the birth situation, some fathers looked at their partner’s behavioural traits, which they knew from their everyday relationship. Doing so helped them assess their partner’s well-being, for example, or provided clues about her need for support. Their familiarity with their partner’s emotional reaction patterns also helped the fathers recognise the limits of their support during the birth. One father stated, ‘So, then the mood tips, so then my wife goes angry when I say that again. […] I know how she reacts emotionally when something doesn’t sit well with her’ (P22).

On the other hand, the discrepancy experienced between their wife’s behaviour in everyday life and the ongoing birth process could be perceived as unsettling. One participant, for example, found his partner’s otherwise unusual passivity and visible fear challenging and difficult to bear. He explained, ‘And I could see it clearly in her face, there was just pure fear written all over it, and I had simply never seen her like that before. That really unsettled me’ (P7).

### Consequences

Consequences resulted not only from having gained one’s bearings but also from a lack thereof, which could occur despite the action strategies used by the fathers due to insufficient participation or because of care that was perceived as inadequate. Consequences were also expressed at the emotional-affective level of the fathers. The individual consequences presented below included *being able to act*, *accepting the situation*,* knowing that one’s partner was in good hands*,* being helpless and overwhelmed* and *wishing to repeat or avoid experiences next time.*

### Being able to act

The experience of being able to act as a companion and supporter in the birth situation was an essential consequence of the sense of the orientation gained. Options for action could be divided into active and reactive action. The better the fathers understood the situation, the more determined they were to prepare for the next steps. One father stated, ‘It’s like autopilot, how you approach it […]. And then it was clear, okay, we now have to do this, this, this and this, bang, car keys and then driving the car, etc.’ (P4).

When the fathers’ empowerment was also recognised and supported by the professionals, it gave the fathers a sense of participation and of being an equal member of the care team. One participant remarked, ‘Everyone knew what to do. I knew what to do with my wife and what the midwife needed from me. A real team effort’ (P11).

In addition, the fathers’ ability to act contributed to their sense of control in the birth situation. When the fathers experienced the birth as something that they could help shape, this had a positive effect on their well-being and contributed to a positive birth experience. One father explained, ‘With the last birth, I found it so great because I also contributed to it to some extent, which somehow made me very, very satisfied inside. I found it to be quite a great experience’ (P2).

These findings show that the ability to act is not solely a consequence of getting one’s bearings: it can also result from the preparatory strategy for the supportive role. This role was shaped by the expectations of both the partner and the professionals, which further influenced the fathers’ perceived ability to contribute effectively during birth.

### Accepting the situation

Accepting the circumstances of a labour situation that could not be influenced personally was another consequence of getting one’s bearings. For many fathers, the experience of seeing their partner in labour and being unable to relieve her physical pain was a major challenge that they tried to overcome with varying degrees of success. If the fathers could apply the knowledge that they had gained in antenatal classes to the birth situation or could use this knowledge to explain their partner’s behaviour, they were more likely to be able to accept an emotionally challenging situation.

That the contractions are the body’s own, so the way it was told in the course, that’s the way it is, and you can’t take it away from her, and that was okay for me in the situation. I was able to go along with it and come to terms with it. (P4)

### Knowing that one’s partner is in good hands

For the fathers, the well-being of their partner was a key indicator of adequate care by the professionals. Knowing that their partner was in good hands was a result of an assessment of the care provided by the professionals. It was also a confirmation of the trust placed in the caregivers. Knowing that their female partner was in good hands contributed to the fathers’ sense of security and simultaneously enabled them to hand over responsibility to the caregivers. One father stated, ‘I totally trusted the physician and the midwife, and somehow, I was able to hand it over to them. I just knew they would take good care of her and give her what she wanted so much’ (P2).

### Being helpless and overwhelmed

When the fathers were unable to navigate a situation despite the use of orientation strategies, this inability could lead to feelings of helplessness and being overwhelmed. A lack of communication could lead to helplessness and excessive demands, especially when the fathers were increasingly dependent on caregivers for guidance and had no previous experience of childbirth or other knowledge resources to draw on.

Helplessness could also result from a loss of trust in the caregivers. In particular, when the fathers felt excluded and unable to protect their partner from care that was perceived as disrespectful, this feeling reinforced their sense of powerlessness and contributed to a sense of loss of control. In particular, one participant in the study perceived the care provided during childbirth as physically abusive, leaving him feeling completely powerless and at the mercy of the situation as a helpless observer:

We felt like strangers, so we weren’t a group there. It was just the midwives and doctors and us, and we were the victims, so to speak, as if we were anaesthetised. So, in retrospect, it felt like I was watching a rape because I couldn’t do anything. And they talked about it among themselves, but as if we weren’t even there. (P13)

Importantly, this was the participant’s subjective perception, and the study design does not allow us to objectively draw conclusions about the appropriateness of the care.

### Wishing to repeat or avoid experiences next time

The fathers’ approach to future births was characterised by their reflection on their ability to get their bearings and the associated evaluation of their obstetric experience. If the fathers succeeded in getting their bearings in the birth situation, they preferred the same birth setting or the same caregivers for a potential subsequent birth to repeat the positive experience. If the fathers did not find their way in a particular situation despite their orientation strategies, they tried to avoid the experience as much as possible the next time and improve their preparation strategies.

If we go to a hospital again, I would definitely do everything I possibly can to ensure that things unfold the way my wife and I want them to. I would discuss everything with my wife beforehand and inform myself more about our rights. (P13)

## Discussion

The aim of this study was to develop a theory of the constitution of fathers’ sense of security in the context of childbirth. The results demonstrate that getting one’s bearings is a central phenomenon of fathers’ sense of security. To date, the concept of orientation has not received attention in the context of childbirth. Jones [45] described the concept of psychological orientation as an individual interaction process that focuses on successful adaptation to an organisation or environment depending on personal resources and organisational/environmental factors. According to Jones [45], pregnancy and childbirth can be seen as challenging processes that cause fathers to seek orientation to cope with these processes and fulfil their expected roles. In a qualitative study focusing on men’s and healthcare professionals’ constructions of masculinity in relation to pregnancy and childbirth, Dolan and Coe [46] concluded that fathers find themselves in a marginalised position because pregnancy and childbirth are perceived as female domains. The resulting restrictions on their autonomy in an unfamiliar situation makes fathers, particularly first-time fathers, more compliant and in need of guidance [46, 47].

Fathers’ search for orientation includes pregnancy and childbirth and extends beyond birth. Persson et al. [32] argued that fathers’ postnatal sense of security emerges from their participation in and experience of the processes of pregnancy, childbirth, and early parenthood. Therefore, the need to get bearings can be considered a continuous orientation process in the transitional phases to fatherhood, and it corresponds with social-psychological findings regarding a sense of security as an elementary human need [18, 48].

The results of our study show that fathers understand childbirth as an ambiguous and uncertain situation in which they need orientation. The uncertainty and ambiguity of childbirth can lead to insecurity and affect fathers’ understanding of the birth situation and their own role [11, 37, 46, 48, 49]. By preparing themselves, fathers try to reduce their insecurity regarding the birth process by being able to act as a support person. However, preparation not only is self-motivated but also essentially depends on the participation and support of professionals and female partners [28, 32, 34]. Our results show that when professionals recognise fathers’ supportive role and involve them in childbirth, these actions help fathers find their bearings and create a sense of security. In addition, the way professionals communicate and behave plays a crucial role in whether fathers feel validated in their role or uncertain about their involvement. The literature shows that when professionals actively involve fathers in pregnancy and childbirth, fathers’ sense of security is enhanced, helplessness is reduced, and their ability to support the mother is strengthened [28, 34, 37].

In this study, the fathers’ preparation for possible emergency scenarios was essential. It was important for the fathers not only to practise their part in managing an emergency but also to have basic reassurance that the caregivers had the expertise to manage an emergency successfully. The literature shows that birth emergencies are associated with a loss of control for fathers and can lead to traumatic experiences [36, 48, 50]. Our findings confirm that the participants experienced emergency caesarean sections as very stressful. The fathers felt emotionally overwhelmed regardless of their confidence in the competencies of the care team. This acute distress was particularly evident when they were abruptly separated from the event, as they were not allowed in the operating theatre during the procedure. Johansson et al. [36] suggested that the emotional stress associated with an emergency caesarean section stems from fathers’ concern for the mother and child. Similarly, Koppel and Kaiser [51] and Yokote [52] reported that when fathers are left alone and without support during an emergency caesarean section, they exhibit high levels of anxiety and stress.

Our findings show that to get their bearings in changing situations, fathers rely on knowledge and information as essential resources in their mental and practical preparation for childbirth. Previous research has shown that being prepared and receiving information promote fathers’ sense of security [32–34, 49, 53], are associated with a sense of being in control [35–37, 54], and reduce anxiety and vulnerability [49, 55, 56]. However, the participants consciously selected which information to engage with and avoided details that conflicted with their expectations of childbirth or triggered emotional distress. In doing so, they balanced remaining informed with protecting themselves from being emotionally overwhelmed. In addition, our data show that existing trust in the female partner or caregivers influenced the intensity of information seeking. Thus, the perceived sense of responsibility appears to be an influential factor in fathers’ perceptions of their role demands.

This balance, however, is not always easy to achieve. While knowledge and information can provide a sense of preparedness and control, they also have the potential to overwhelm, particularly for first-time fathers. Without prior experience, some of the participants in our study struggled to contextualise the information that they received, which led to feelings of insecurity rather than reassurance. Some of them actively sought knowledge to enhance their sense of control, whereas others deliberately avoided distressing details and feared that too much information might increase their anxiety. Our findings align with those of previous research showing that first-time fathers often navigate the tension between the desire to be informed and the risk of emotional overload [47]. Similarly, while access to information is generally appreciated, moments of emotional strain can arise, particularly when unexpected complications occur [56]. The findings above indicate that the role of information in shaping fathers’ experiences is highly individual and is influenced by prior exposure, personal coping strategies, and trust in caregivers.

Previous findings characterised a sense of security as a multidimensional concept created along emotional, social, and physical-medical dimensions [27, 33]. In line this perspective, our findings indicate the emotional dimension of a sense of security in fathers’ trust in professionals, confidence in women’s childbearing competence and their own supportive skills. Furthermore, partnerships had an impact on the emotional experience of fathers. Our study revealed fathers’ emotional attachment to their partners from the familiar ‘we’ perspective; almost all the interviewees switched to this perspective in their narratives. In addition, our findings indicate that mothers’ well-being contributes to fathers’ orientation and, thus, their sense of security, which is in line with the findings of quantitative and qualitative studies focusing on the sense of security in the postpartum period [28, 31, 32].

Conversely, mothers’ discomfort or helplessness can lead to insecurity and emotional crisis in fathers. Persson and Kvist [29] concluded that a low level of security in both parents in the postpartum phase was causally related to stress and the risk of postpartum depression. Furthermore, helplessness and powerlessness seem to lead fathers to experience emotional conflict regarding their male identity [46, 57].

All of these emotions illustrate the vulnerability of fathers. In the literature, vulnerability during childbirth is frequently associated with contradictory feelings, such as euphoria, happiness, fear, powerlessness, inner stress, and an inability to act, which fathers may experience individually or simultaneously at different points throughout the childbirth process [11, 49, 56, 58]. None of the study participants confessed their emotional distress to their partner or professionals, as they considered their feelings, especially during childbirth, to be unimportant to the situation. These findings align with those of previous research suggesting that fathers may perceive the woman’s physical pain during labour as more legitimate than their own emotional distress, a perception that can be reinforced by professionals [46, 57, 59]. Dolan and Coe [46] argued that men hide their emotional distress to avoid appearing weak and selfish because of culturally idealised forms of masculinity that construct men as stoic and self-reliant.

The physical-medical dimension of a sense of security was demonstrated in our study by fathers’ desire for the safe care of the mother and child and professionals’ skills, as confirmed in several studies [28, 32–34]. For expectant fathers, the health of the mother and child is the highest priority [60]. In this study, it was not only the actual risks that were important for fathers but also the potential risks that were influenced by their risk-oriented perceptions of childbirth.

The potential risks were used by the fathers in our study to negotiate their partner’s wishes regarding the place of birth and, in some cases, to mark the limits of acceptance of their partner’s needs and wishes. Robson et al. [61] reported that the perception of risk differs between men and women with respect to the mode of birth. The strategy of risk assessment and weighing in favour of the lowest risk can be seen as an attempt by fathers to control the potential health risks for the mother and child. This strategy is in line with the guiding principle of sociological risk research: the lower the risk is, the greater the objective safety [62, 63].

The fact that fathers’ ability to orient themselves depends on their partners and professionals can be considered part of the social dimension of security. Fathers’ perceptions of safety during childbirth are shaped not only by personal experiences but also by the medicalisation of childbirth and gendered expectations of paternal responsibility, both of which influence fathers’ decision-making and coping strategies [11, 36]. A broader perspective on these social influences is essential for understanding why many fathers associate safety primarily with a clinical setting [1, 36]. Likewise, their emphasis on risk assessment and the prioritisation of safety over personal or partner preferences may reflect gendered norms that position men as responsible for ensuring control in uncertain situations [14]. At the same time, ideals of dominant masculinity discourage fathers from expressing distress, as emotional restraint aligns with societal expectations of male strength and self-reliance [1, 11, 46].

Therefore, the different dimensions of fathers’ sense of security are not considered separately. The emotional dimension of fathers’ sense of security can be seen as a result of the social and physical-medical dimensions.

One potential limitation of this study is that the sample was not demographically representative, as most of the fathers who agreed to participate were highly educated and employed. The fathers’ previous experience of childbirth was another limitation, as the factors that affected their sense of security differed between first-time fathers and experienced fathers. Moreover, the findings are based on the experiences of fathers who were present at birth. It remains unclear whether the processes to achieve a sense of security also apply to fathers who did not accompany their partners during childbirth. In addition, all but one of the participants were German; thus, the findings may not reflect the experiences of fathers from other cultures. Another potential limitation is the timing of the interviews. Both parents were interviewed simultaneously approximately 6 months after the last birth, but they were interviewed separately. It was decided to conduct the interviews at this time because both parents should have had time to adjust to parenthood and to their maternal and paternal roles [64]. Even though almost all the participants recalled emotional situations during childbirth and reproduced their memories accurately and extensively, recall bias cannot be ruled out. Despite these limitations, this study explored the constitution of fathers’ sense of security in the context of childbirth and identified the relationships between security-creating factors, including conditions that influence the phenomenon, action strategies, and consequences.

## Conclusion

The findings of this study indicate that fathers’ sense of security is constituted by their orientation towards their partners and professionals as well as the birth situation. The results underline the importance of fathers’ involvement in pregnancy and childbirth. Additionally, the empowering behaviour of midwives and obstetricians is essential for fathers to experience a sense of security during pregnancy and childbirth. Professionals working in antenatal and postnatal services should discuss possible emotional challenges with fathers and create opportunities for fathers to express their own need for security. There is also a need for further research on professional caregivers’ perceptions of fathers’ security needs to support fathers effectively during pregnancy and childbirth.

## Data Availability

The data are not publicly available due to privacy or ethical restrictions. However, the data that support the findings of this study are available from the corresponding author upon reasonable request.
